# Reciprocal communication between mesenchymal stem cells and leukemic cells: role of junctional complexes and exosomes in adult T-cell leukemia progression

**DOI:** 10.1186/1742-4690-12-S1-P59

**Published:** 2015-08-28

**Authors:** Jamal El-Saghir, Tala Kanson, Farah Kouzi, Marwa Hussein, Marwan El-Sabban

**Affiliations:** 1 Department of Anatomy, Cell Biology, and Physiological Sciences, Faculty of Medicine, American University of Beirut, Lebanon; 2 Department of Biology, Faculty of Sciences & DSST, Lebanese University, Lebanon

## Introduction

A specialized microenvironment in the bone marrow niche is composed of stromal cells including mesenchymal stem cells (MSCs) that might support leukemia progression. In this study, we investigated the reciprocal interaction between MSCs and Adult T-cell Leukemia (ATL) cells, as a model of hematological malignancies. We evaluated the role of paracrine interaction between MSCs and leukemic cells through soluble factors, exosomes, and direct interaction through adhesion (N-cadherin) and communication (Connexins).

## Methods

ATL cells used were either HTLV-I negative (Molt-4) or positive (C81 and HuT-102) cells. Indirect co-cultures between MSCs and ATL cells were assessed in Transwell chambers whereby MSCs were seeded in the bottom well and leukemic cells were in the upper insert. Direct co-cultures were performed at 1:1 ratio and cells were then sorted using CD73, MSCs’ cell surface marker. Leukemic cells-derived exosomes were isolated by ultracentrifugation and co-cultured with MSCs. Following co-cultures for 72 hours, we studied cell proliferation by Trypan Blue Exclusion assay and expression profile of metastatic and stemness markers by Real-time PCR and western blotting.

## Results

After indirect co-culture, MSCs caused a moderate increase in ATL cells proliferation. In contrast, MSCs proliferation was only induced by HuT-102 cells. Although indirect co-culture did not cause major changes in leukemic cells expression profile of metastatic markers, direct interaction induced the expression of Cx43, SDF-1 and VEGF, especially in the adherent fraction, compared to the suspension fraction. Exosomes derived from C81 and HuT-102, HTLV-I positive cells, contained Tax oncoprotein and induced MSCs proliferation. This interaction led to an increase in MSCs cell number, a change in cellular morphology and an increase in the expression of VEGF and stemness markers, Oct-4 and Nanog.

## Conclusion

These findings demonstrate that indirect and direct interactions reciprocally affect MSCs and leukemic cells properties. Interaction through leukemia-derived exosomes modulates MSCs properties which might in turn contribute to leukemia progression.

